# Promoting equity, diversity and inclusion in randomised trials in Australia: designing a think tank using aspects of World Café and Yarning Circle methodologies

**DOI:** 10.1186/s13063-026-10046-5

**Published:** 2026-11-03

**Authors:** Mark Liu, Bec Jenkinson, Lorraine Tutton, Kate Aitchison, Nadine E. Foster, Nathalia Costa, Elise Strange, Ingrid J. Hickman

**Affiliations:** 1 ULTRA (The University of Queensland’s Randomised Trials Capability), Faculty of Health, Medicine and Behavioural Sciences, The University of Queensland, Brisbane, Australia; 2 Gamilaraay/Murawari Aboriginal Woman, Consumer Representative, Brisbane, Australia; 3 STARS Education and Research Alliance, Surgical Treatment and Rehabilitation Service (STARS), The University of Queensland and Metro North Health, Brisbane, Queensland Australia; 4 Cancer and Palliative Care Outcomes Centre, Faculty of Health, Queensland University of Technology, Brisbane, Australia

**Keywords:** Equality, Intersectionality, Representation, Disparities, Stakeholder engagement, Consumer and community involvement, Patient and public involvement, Ethics, Governance

## Abstract

**Background:**

Equity, diversity and inclusion in randomised trials is widely acknowledged as a scientific and ethical imperative, yet persistent barriers remain relating to cultural, linguistic, geographical and other social and structural determinants of health. Such determinants become pervasive systemic barriers that compromise the real-world relevance of randomised trials and perpetuate health inequities. International and national Australian regulatory bodies promote equity, diversity and inclusion in randomised trials, but implementation is inconsistent. This commentary describes the methodological process to design and conduct a think tank event as a strategy to raise awareness and promote the consideration of equity, diversity and inclusion in randomised trials in Australia.

**Main body:**

Through outreach via university and clinical research networks, we identified and invited 27 attendees to a think tank event that explored strategies for advancing equity, diversity and inclusion in Australian randomised trials. Held in Brisbane, Queensland, attendees included academics, clinician researchers, social impact and cultural diversity professionals, research ethics and governance professionals, consumer and community representatives, and other research-adjacent service providers. The event drew upon two participatory discussion methodologies, World Café and Yarning Circle, to facilitate in-depth conversation amongst the diverse attendees. Discussions flowed from three guiding questions regarding randomised trials in Australia: “What does health equity mean?”, “what factors influence health equity?”, and “what are the next steps?” Combining World Café’s structured, idea-generating format with Yarning Circle’s culturally grounded, relational approach enabled both breadth and depth of insights. Insights generated from the think tank event were graphically recorded by a professional artist, which will be combined with other collected data (online and table notes) to inform future actions.

**Conclusion:**

Addressing equity, diversity and inclusion in randomised trials requires moving beyond policy statements to practical, collaborative action. Participatory methods such as World Café and Yarning Circle are favourable approaches for fostering inclusive dialogue and co-designing ideas that can inform future action.

## Background

Whilst randomised trials are widely considered the most rigorous research method to determine the comparative effectiveness of health and medical interventions, their traditional emphasis on high internal validity has meant that the findings of many randomised trials are not then generalisable beyond the research context and the participants [1]. Inclusion criteria such as language proficiency, proximity to metropolitan trial sites, arbitrary age cut-offs and/or the presence of co-morbidities are commonly applied to facilitate participant homogeneity and ease trial conduct. However, if such criteria are in place without scientific justification, they become unjustified barriers to participation and systematically exclude groups experiencing the greatest health inequities, with the consequence that the results of such trials have, at best, unclear relevance to real-world communities [2–4]. Whilst a continuum exists between pragmatic trials (valuing external validity) and explanatory trials (valuing internal validity) [5], the trial sector generally tends to rely on entrenched practices that prioritise convenience and control, over equity and real-world applicability. At the forefront of evidence generation, the trials sector has been described as a “canary in the coal mine”, suggesting that inequities that occur in randomised trials are likely to be perpetuated as implementation of evidence into healthcare settings occurs [6].

Acknowledging the inequities in randomised trials research, many regulatory bodies assert that healthcare research must serve the communities it intends to benefit. At the international level, the Declaration of Helsinki states that “groups that are underrepresented in medical research should be provided appropriate access to participation in research” [7]. This is echoed by the International Council for Harmonisation, stipulating that “the participant selection process should be representative of the population groups that the investigational product is intended to benefit… to allow for generalising the results across the broader population” [8]. In the Australian context, the National Statement on Ethical Conduct in Human Research is aligned to these principles: “researchers have an obligation to design and conduct research in such a way that opportunities to participate in research are equitable and as inclusive as possible” [9]. These principles are not only policy and ethical imperatives, but also key practical recommendations to enhance external validity and reduce research wastage [10].

Despite these equitable, ethical, consumer and community-centred guidelines that ostensibly underpin the randomised trials sector, the concepts of equity, diversity and inclusion remain a notable policy-implementation gap [11]. This commentary paper outlines the strategies we undertook to explore innovative strategies to raise awareness and promote equity, diversity and inclusion in randomised trials in the Australian context. An initial outreach phase was conducted that led to a think tank event applying participatory methods that are novel to the topic and attendees: combining aspects of the World Café with Yarning Circle methodologies to facilitate dialogue around promoting equity, diversity and inclusion in randomised trials amongst a diverse group of interest-holders. The aim was to reflect on the appropriateness of the think tank methods for collecting constructive information and facilitating future collaborations amongst the attendees.

## Initial outreach

As an initial step, we undertook extensive outreach engagement through university and clinical research networks and peer referral to meet and discuss with researchers experienced in equity, diversity and inclusion and/or randomised trials. These occurred either face-to-face at a place convenient to the contributor or online via a video conference, for approximately 30–60 min. Researchers who engage with equity, diversity and inclusion topics in the Australian trials sector often work within domains focused on particular population groups, for example, Australian Aboriginal and Torres Strait Islander peoples, regional, rural and remote communities, multicultural communities, people with cognitive or communication barriers, older adults, children and adolescents. Whilst each domain has unique considerations for promoting equity, diversity and inclusion in randomised trials, there are overarching systemic factors that influence equity, diversity and inclusion across all randomised trials broadly (inclusive of investigator or industry-led trials, for therapeutic and health service interventions). As the outreach discussions occurred, some examples of overarching factors that were repeatedly raised included inconsistent participant demographic data, recruitment and consent challenges, the training needs of randomised trial teams, and the education needs of consumers and communities [12–14].

During this outreach, it was recommended that discussions should extend beyond researchers to include other interest-holders across the health research and randomised trials sector, such as research ethics committee members, research governance officers, funders, and consumer and community representatives. We hence broadened our approach, ultimately conducting >60 conversations with individuals across multiple Australian states and territories (Queensland, New South Wales, Victoria, South Australia and the Australian Capital Territory) and one international contributor. During this process, an Australian Aboriginal collaborator (LT) raised the value of incorporating aspects of traditional Yarning Circle methodology as part of future activities, to bridge Indigenous Australian and Western ways of knowing and sharing.

## The think tank

Building upon the insights from the initial outreach discussions, we identified the need for an in-person event to consolidate the informal discussions and to create a dedicated space and time for deeper and collective dialogue. A face-to-face format was also considered advantageous for seeding collaboration for future work. For logistical reasons, the event was based locally in one state in Australia (Queensland), with invitations extended to attendees from regional, rural and remote localities (travel costs were supported, and consumers were remunerated for their time). The think tank event was structured around three broad, core questions regarding randomised trials:

1. What does health equity mean?
2. What factors influence health equity?
3. What are the next steps?

The values and design principles from two methodologies were suggested as particularly suitable for the aims of event and the topic of equity, diversity and inclusion: the World Café and Yarning Circle.

### World café

The World Café method, originating from the mid-1990s in the USA, is a structured group discussion process designed to foster knowledge sharing that leads to collaborative dialogue [15–17]. Attendees begin in small-group discussions centred on a specific question, with key ideas captured and shared as participants rotate between tables. As the questions progress, people move between groups and report back on previous conversations, so that ideas are progressively refined, expanded and challenged. This allows diverse perspectives to cross-pollinate, and collective insights to emerge. The approach is founded on the idea that meaningful insights often emerge through conversation and shared reflection rather than individual standalone contributions. This method is particularly suited to complex topics, such as equity, diversity and inclusion in trials, as it [18]:

- Encourages a relaxed, hospitable atmosphere purposely designed to reduce power imbalances and encourage candid discussion.
- Generates collective insights rather than isolated opinions.
- Enables ideas to emerge organically through iterative dialogue.

For the randomised trials sector, where equity, diversity and inclusion challenges span across clinical fields and systems layers, drawing upon World Café principles allows for multiple perspectives to be incorporated together from attendees from diverse professional and personal backgrounds.

### Yarning circle

Yarning Circles are an Indigenous Australian approach to dialogue that is grounded in respect, listening and trusting relationships [19, 20]. It involves the exchange of knowledge through sharing stories, experiences or perspectives, along with listening in a respectful and reflective manner, in order to build mutual learning. The approach foregrounds equal voices and a safe environment, so that everyone has the opportunity to yarn without feeling pressured or judged. Yarning has existed for millennia, and comparable practices are mirrored in other Indigenous cultures, for example, amongst Native American peoples [21]. It emphasises storytelling as a way of exchanging ideas and building respect between participants. This approach is suitable and advantageous for equity, diversity and inclusion discussions because it:

- Creates a culturally respectful environment for all voices, including Australian Aboriginal and Torres Strait Islander perspectives.
- Values lived experience alongside professional expertise.
- Promotes deep, empathetic listening, which is essential when discussing health and other related inequities.

In the context of an equity, diversity and inclusion-themed event in randomised trials, incorporating values from the Yarning Circle methodology ensures that conversations are inclusive and foster trust between attendees, allowing for more honest and meaningful sharing. Although of an Indigenous Australian origin, the underpinning relational principles of trust-building and storytelling may also be beneficial when discussing health equity in other contexts as well [22].

### Complementary strengths

The program of the think tank event, therefore, employed the discussion steps and methods of a World Café, and the Indigenous values of Yarning Circles were foregrounded at the start of the day. Combining the World Café’s structured, idea-generating format with Yarning Circles’ culturally grounded, relational approach allowed the think tank event to balance productivity with conversational richness (Table 1).

**Table 1:** Comparison of facilitation methods that informed the think tank event

	World Café	Yarning Circle
Purpose	Structured dialogue to generate collective ideas and insights	Relational dialogue to build trust and share personal experiences
Format	Rotating small-group discussions around key questions	Circle-based storytelling with emphasis on equal voices and listening
Strengths	Encourages breadth of perspectives	Encourages depth of understanding
Suitability for discussing equity, diversity and inclusion	Surfaces common issues across diverse attendees	Surfaces personal reflections

### Attendees

Twenty-seven attendees took part in the 1-day think tank event in Brisbane, Queensland, Australia, in November 2025. Attendees included:

- Those with experience engaging with historically underserved communities, in randomised trials or other methods (e.g. Indigenous Australian peoples, regional/rural/remote communities, multicultural communities, people living with disability).
- Academics and clinician researchers (ranging from early-career to professorial).
- Social impact and cultural diversity professionals.
- Research ethics and governance professionals.
- Consumer and community representatives.
- Interpretation and translation service providers.

### Capturing the dialogue

Conversations were captured using multiple approaches. An online interactive platform (Mentimeter.com) was used in parallel to table notes (butchers'/flipchart paper and sticky notes) to gather and visually organise data across the three questions. A socioecological “onion” model for the randomised trials sector was introduced as a simple approach for organising ideas according to system layers (i.e. individual, inner and outer contexts) [23]. Additionally, a professional artist was engaged to make graphic recordings throughout the think tank event (artbalm.com). Lastly, an evaluation survey was emailed to attendees the following day, asking for their key takeaways, intended next actions, feedback and any other suggestions. Thematic and systems mapping analyses will be applied to the collected data, which is then intended to inform future trial methods and research questions.

**Fig. 1:**
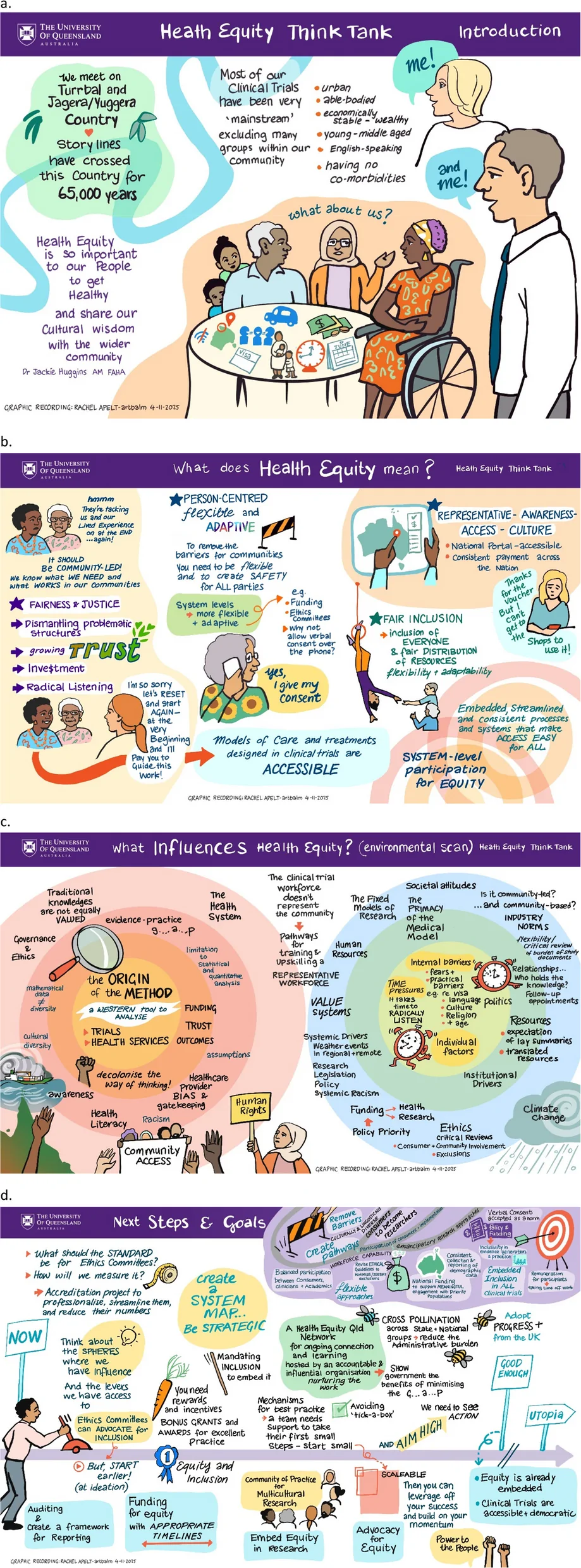
Graphic recordings of the think tank event (graphic recorder: Rachel Apelt—Artbalm) [Alt text: A visual summary of discussions about health equity in randomised trials in Australia. There are four panels: introduction of the topic, defining the concept of health equity, describing the influential factors and suggesting future actions.]

The graphic recordings are shown in Fig. 1a–d. In summary, Fig. 1a is an overview of the event scope and purpose, highlighting the need for promoting equity, diversity and inclusion in randomised trials. Figure 1b is a graphic summary of discussions relating to the first question (what does health equity mean?): topics included person-centredness principles of promoting trust between consumers and health/research professionals, and avenues for making trial processes more streamlined and flexible to reduce barriers to participation. Figure 1c is a graphic summary of discussions relating to the second question (what factors influence health equity?): topics included the pervasive barriers of time and funding shortages across all systems levels, and the suitability of traditional randomised trial epistemologies for contemporary social factors. Figure 1d is a graphic summary of discussions relating to the third question (what are the next steps?): topics included short-term actions specific to attendees’ spheres of influence (establishing networks, adapting guidelines from other countries for Australian context, supporting small changes within local teams) and medium to long-term actions to facilitate sustained advocacy efforts (incentives and mandates facilitated through funding agencies and ethics committees).

Through the emailed online survey, attendees were given the opportunity to share their thoughts on potential next steps from the think tank event, as well as any feedback and suggestions. Responses included planning to follow up with new contacts, disseminate summaries from the day, review their own projects for opportunities to elevate equity, diversity and inclusion, and explore more inclusive research practices, particularly around engaging under-represented groups and collecting more comprehensive demographic data. Continued collaboration was desired, potentially forming an ongoing working group. Feedback on the event format was positive: attendees valued the participatory structure, deep discussions and real-time visuals from the graphic recorder and online interactive platform. Suggestions included involving additional and more diverse interest-holders and maintaining momentum through future events.

Table 2 provides a stepped summary of the activities that took place in order to design and implement the think tank.

**Table 2:** Summary of think tank implementation steps

Step	Activity description
Initial outreach	• Identify and meet with subject matter experts, locally and interstate (online) • Snowball the outreach through referrals and suggestions • Consider representativeness across sociodemographic groups, as well as clinical, professional and/or consumer background
Design	• Identify the need for an in-person group event for the cross-pollination of ideas and networking for future activities • Meet with all potential attendees beforehand to ensure that there is a pre-established, shared understanding of the premise and desired outcomes prior to the day • Consider event format, in partnership with the planned attendees, e.g. the complimentary nature of structured groups and questions (World Café principles) combined with an emphasis on authentic relationship-building (Yarning Circle principles) • Share or discuss the next steps upfront to build long-term collegiality
Logistical considerations	• Consider a “neutral ground” venue that is not affiliated with an organisation, to reduce perceptions of power or ownership of resulting ideas • Create a welcoming and comfortable environment to encourage open sharing (e.g. décor, catering) • Have multiple approaches to record ideas, both for triangulation and redundancy purposes, which can be done by attendees themselves as well as a dedicated facilitator(s)
Potential constraints and solutions	• As per the World Café guidance, the size and total number of small groups can vary depending on the available time and complexity of questions. Consider the balance between having a sufficient diversity of perspectives and keeping groups small enough to let everyone contribute meaningfully • It may be challenging for attendees to commit to a whole-day, uninterrupted event. Allow enough time between invites and the event day and prioritise inviting attendees who are personally committed to actively participating and contributing • Differences in personalities and communication styles may lead to some attendees to contribute more than others. Consider spreading formal or informal facilitators across groups to encourage balanced discussion and ensure that everyone has an opportunity to contribute • Concepts from the World Café and Yarning Circle rely on the competency of both the organisers and attendees. It is helpful to involve or learn from individuals with previous experience and have pre-event one-on-one meetings with attendees to set expectations

## Conclusions

Advancing equity, diversity and inclusion in randomised trials requires moving beyond aspirational policy statements towards practical, collaborative action. Our experience conducting this think tank event demonstrates that creating space for inclusive dialogue, where diverse interest-holders can share perspectives and co-design ideas for proposed solutions, is appropriate and effective for collecting constructive information and facilitating new connections for future collaborations on equity-related activities. Participatory methodologies like the World Café and Yarning Circle offer practical and powerful tools for this type of engagement work. The World Café provides structure and efficiency, enabling broad idea generation across disciplines, whilst the Yarning Circle methodology ensures cultural responsiveness and relational depth, fostering trust and valuing lived experience. Together, these methods create the conditions for meaningful engagement and actionable insights.

Whilst long-term impact requires continuous engagement, this think tank event represents a step towards advancing the goal of randomised trials serving all communities, thereby strengthening the relevance and impact of health and medical research for all.

## Data Availability

No datasets were generated or analysed during the current study.
